# Therapeutic Potential of Alpha-Lipoic Acid: Unraveling Its Role in Oxidative Stress and Inflammatory Conditions

**DOI:** 10.3390/cimb47050322

**Published:** 2025-04-30

**Authors:** Aqsa Shahid, Khadeeja Nasir, Madhav Bhatia

**Affiliations:** 1 Department of Pathology and Biomedical Science, University of Otago, Christchurch 8140, New Zealand; shaaq042@student.otago.ac.nz; 2 Department of Medical Laboratory Technology, Faculty of Allied Health Sciences, Superior University, Lahore 54000, Pakistan; khadeeja.nasir@superior.edu.pk

**Keywords:** alpha-lipoic acid, dihydrolipoic acid, anti-inflammatory agent, antioxidant agent, hydrogen sulfide (H_2_S)

## Abstract

Alpha-lipoic acid (ALA) is an essential organosulfur compound with a wide range of therapeutic applications, particularly in conditions involving inflammation and oxidative stress. In this review, we describe our current understanding of the multifaceted role of ALA in several inflammatory diseases (acute pancreatitis, arthritis, osteoarthritis, asthma, and sepsis), cardiovascular disorders (CVDs), and neurological conditions. The dual redox nature of ALA, shared with its reduced form dihydrolipoic acid (DHLA), underpins its powerful antioxidant and anti-inflammatory properties, including reactive oxygen species scavenging, metal chelation, and the regeneration of endogenous antioxidants such as glutathione. A substantial body of evidence from preclinical and clinical studies suggests that ALA modulates the key signaling pathways involved in inflammation and cellular stress responses, making it a promising candidate for mitigating inflammation and its systemic consequences. Notably, we also discuss a novel perspective that attributes some of the therapeutic effects of ALA to its ability to release hydrogen sulfide (H_2_S), a gaseous signaling molecule. This mechanism may offer further insights into the efficacy of ALA in the treatment of several diseases. Together, these findings support the potential of ALA as a multifunctional agent for managing inflammatory and oxidative stress-related diseases.

## 1. Introduction

Alpha-lipoic acid (ALA), a naturally occurring dithiol compound, is a potent antioxidant and anti-inflammatory agent with critical roles in cellular energy metabolism as a cofactor for mitochondrial dehydrogenase complexes. Its unique ability to act as both a lipid- and water-soluble antioxidant enables it to scavenge reactive oxygen species (ROS), regenerate endogenous antioxidants (e.g., glutathione and vitamin C), and modulate the key signaling pathways implicated in oxidative stress and inflammation. While only small amounts of ALA are synthesized endogenously, it is widely utilized as a dietary supplement owing to its anti-aging, anti-diabetic, and neuroprotective properties. Notably, preclinical and clinical studies have demonstrated its efficacy in improving cognitive impairment and mitigating age-associated neuromuscular degeneration [1,2]. The dysregulation of oxidative stress and inflammatory responses represents a hallmark of numerous pathological conditions, including sepsis, acute pancreatitis, asthma, joint inflammation, cardiovascular diseases (CVDs), and neurological disorders. Uncontrolled inflammation and aberrant immune activation further exacerbate tissue damage and disease progression, underscoring the need for therapeutic strategies targeting these interconnected pathways. This review provides a comprehensive analysis regarding the important role of ALA in sepsis, acute pancreatitis, joint inflammation, CVDs, and neurological diseases, with a focus on its molecular mechanisms of action, therapeutic potential, and translational relevance across experimental and clinical settings.

## 2. Properties of Alpha-Lipoic Acid (ALA)

Alpha-lipoic acid (ALA), a potent organosulfur antioxidant derived from caprylic acid (octanoic acid), exhibits numerous health benefits. First isolated in 1951, this chiral compound (also known as thioctic acid or 1,2-dithiolane-3-pentanoic acid) plays a critical role in mitochondrial energy production [3]. ALA is efficiently absorbed in the gut, crosses the blood–brain barrier (BBB), and not only functions as a direct antioxidant but also enhances the activity of other antioxidants, such as vitamins C and E, while boosting glutathione synthesis [4]. It regulates glycine synthesis and degradation in mitochondria and serves as a cofactor for two key mitochondrial enzyme complexes: the α-ketoglutarate dehydrogenase complex (KDC) and pyruvate dehydrogenase complex (PDC). These complexes are essential for glucose metabolism and ATP generation [5].

**Figure 1:**
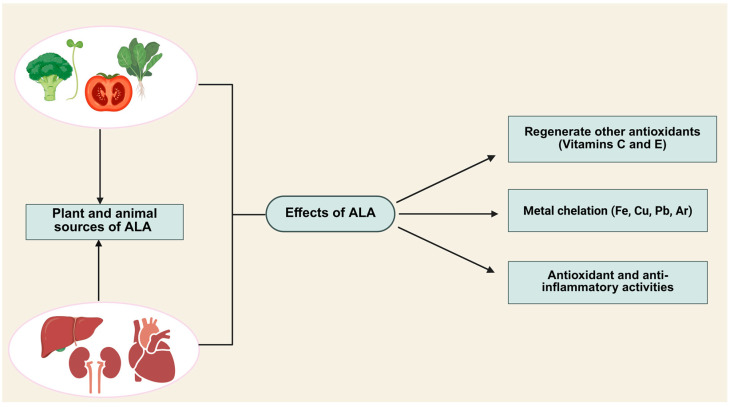
ALA can be obtained from different sources, and it has several functions, such as the regeneration of other antioxidants, the chelation of metals, and anti-inflammatory and antioxidant activities.

The unique amphiphilic nature (solubility in both hydrophilic and lipophilic environments) of ALA supports its systemic distribution and BBB penetration [6,7]. It exists in two optical isomers (S- and R-enantiomers), though only the naturally occurring R-ALA form binds covalently to lysine residues via amide linkages, acting as a cofactor in vital biological processes [8]. Under physiological conditions, ALA is reversibly reduced to dihydrolipoic acid (DHLA). Both oxidized ALA and reduced DHLA are potent natural antioxidants that mitigate oxidative stress by scavenging reactive oxygen species (ROS) [2]. Endogenous ALA is predominantly found in the liver, kidneys, and heart [9], while dietary sources include spinach, peas, broccoli sprouts, and tomatoes [10]. As a dietary supplement, ALA is administered orally, and as a therapeutic agent, it can be administered intravenously (IV). Oral ingestion leads to rapid absorption in the small intestine, with peak plasma concentrations achieved within 30–60 min [11]. Liquid formulations enhance bioavailability compared to tablets, offering faster absorption, higher plasma concentrations, and improved stability [12]. Consuming ALA on an empty stomach (under acidic pH) optimizes gastric mucosal absorption [10], whereas food intake reduces bioavailability due to delayed gastric emptying and interactions with dietary components [11]. Following absorption, ALA binds to albumin for systemic distribution. ALA has been studied for its roles in weight management, appetite regulation, and biomolecule metabolism [13]. Furthermore, a novel lipophilic derivative of ALA, known as octacosanol lipoate, was synthesized, which significantly increased the lipid solubility of ALA and exhibited stronger antioxidant activity [14,15]. Figure 1 summarizes the different sources and effects of ALA. 

## 3. ALA as an Antioxidant

ALA is unique among antioxidants due to its ability to function effectively in both oxidized (ALA) and reduced (DHLA) forms [16]. While DHLA exhibits stronger antioxidant properties, the ALA/DHLA system forms a potent redox pair (standard reduction potential: −0.32 V). DHLA plays a critical role in scavenging free radicals and regenerating endogenous antioxidants such as ascorbic acid (vitamin C) and α-tocopherol (vitamin E) [17]. Additionally, the ALA/DHLA pair inhibits protein carbonyl formation by neutralizing hypochlorite [18] and effectively quenches hydroxyl radicals (•OH) and hypochlorous acid (HClO), though activity against hydrogen peroxide (H_2_O_2_) is limited [6,19]. The low molecular weight and amphiphilic nature of ALA/DHLA enable antioxidant interactions across cell membranes and cytoplasmic compartments, enhancing intracellular antioxidant defense. Key factors determining antioxidant efficacy include metal chelation, free radical scavenging selectivity, bioavailability, gene expression regulation, synergy with other antioxidants, and capacity to repair oxidative damage. All these properties are exemplified by the ALA/DHLA redox pair [16]. Notably, DHLA outperforms ALA in inhibiting lipid peroxidation, a major driver of membrane structural disruption and barrier dysfunction [7,20]. Experimental studies demonstrate the ability of DHLA to scavenge peroxyl radicals in both lipophilic and hydrophilic environments, even in the absence of glutathione (GSH) and vitamin E. It also regenerates ascorbate by reducing ascorbyl radicals and tocopheroxyl radicals [7]. ALA/DHLA further mitigates oxidative stress by chelating redox-active metals. ALA binds copper (Cu^2+^), zinc (Zn^2+^), lead (Pb), manganese (Mn), and arsenic (As), while DHLA chelates ferric (Fe^3+^), cadmium (Cd^2+^), and mercuric (Hg^2+^) ions [21]. These properties underpin ALA’s neuroprotective effects in 6-hydroxydopamine (6-OHDA)-induced Parkinson’s disease mouse models, where it regulates iron metabolism and reduces oxidative stress [22]. Similarly, the ALA/DHLA system alleviates Pb-, Cd-, and As-induced toxicity in PC12 and Caco-2 cells by enhancing intracellular antioxidant activity, activating the Nrf2 signaling pathway, and restoring endogenous GSH levels [23]. Beyond direct effects, ALA/DHLA indirectly supports antioxidant defenses by promoting the uptake of exogenous antioxidants, restoring endogenous antioxidants (e.g., ascorbic acid and α-tocopherol), upregulating antioxidant enzymes, and improving the GSH/GSSG ratio [24]. Intravenous ALA administration elevates GSH levels in LPS-induced kidney injury models [25], and ALA acts as a transcriptional regulator for genes involved in GSH synthesis and homeostasis [26]. Collectively, these attributes position ALA as an ideal and multifaceted antioxidant.

## 4. Role of ALA in Inflammatory Diseases

Inflammation is the protective response of the body towards harmful stimuli such as infection, physical trauma, or chemical injury. During inflammation, proinflammatory mediators are released to activate immune responses aimed at eliminating injurious agents and initiating tissue healing [27]. However, excessive oxidative stress plays a pivotal role in driving the chronic progression of inflammatory disorders. At the cellular level, ALA modulates the NF-κB signaling pathway. NF-κB is a transcription factor critical for the expression of cytokines, chemokines, inducible nitric oxide synthase (iNOS), and adhesion molecules, which are the key drivers of inflammatory processes [28].

This section summarizes the anti-inflammatory effects of ALA across several inflammatory diseases, including acute pancreatitis, arthritis, sepsis, and asthma, with a focus on its mechanistic interplay with oxidative stress and immune signaling pathways.

### 4.1. ALA and Acute Pancreatitis

Acute pancreatitis (AP), as defined by the revised Atlanta classification, is an inflammatory disorder characterized by serum amylase or lipase levels ≥ 3 times the upper limit of normal levels, severe belt-like abdominal pain, and confirmatory radiological imaging findings [29]. The most common etiological factors include gallstones and excessive alcohol consumption [30]. In a cholecystokinin (CCK)-octapeptide-induced AP rat model, ALA demonstrated protective effects by significantly reducing serum lipase and amylase levels, as well as the pancreatic weight-to-body weight ratio. However, no significant alterations in proinflammatory mediators such as TNF-α, IL-1β, and IL-6 were observed [31]. Another study in cerulein-induced AP rats revealed that ALA administration attenuated pancreatic damage through multiple mechanisms: it increased glutathione levels, decreased serum amylase and lipase levels, reduced malondialdehyde (a lipid peroxidation marker) levels, and suppressed myeloperoxidase activity (an indicator of neutrophil infiltration). Histological analysis further confirmed reduced necrosis and mitigated pancreatic tissue injury [32]. In vitro studies using pancreatic acinar AR42J cells exposed to cerulein/resistin-induced oxidative stress and inflammation demonstrated that ALA alleviates these effects by activating the peroxisome proliferator-activated receptor gamma (PPARγ) pathway. PPARγ activation downregulated IL-6 expression and ROS production while upregulating antioxidant enzymes such as heme oxygenase-1 (HO-1) and catalase [33]. Collectively, these preclinical findings suggest the potential of ALA as a therapeutic agent for managing AP.

### 4.2. ALA and Joint Inflammation

Inflammatory arthritis, a hallmark of rheumatic diseases, is most commonly exemplified by rheumatoid arthritis (RA), a debilitating condition characterized by joint destruction, chronic synovitis, pain, and disability [34]. The therapeutic effects of ALA were investigated in a collagen-induced arthritis (CIA) mouse model. ALA treatment suppressed NF-κB activation and reduced the levels of proinflammatory mediators in serum and joint tissues. Additionally, it markedly attenuated bone destruction and inhibited the production of tartrate-resistant acid phosphatase (TRAP)-positive osteoclasts [35]. In another study using CIA mice, ALA administration significantly mitigated pathological bone changes and intracellular ROS levels in lymph nodes. It also suppressed IL-6, TNF-α, and IL-1β expression, as well as NF-κB binding activity, in synovial fluid [36]. Further research explored the effects of ALA on osteoclastic bone damage in inflammatory mouse models. ALA significantly inhibited the receptor activator of nuclear factor kappa-B ligand (RANKL) expression and prostaglandin E_2_ (PGE_2_) production in osteoblast co-cultures. DHLA directly suppressed PGE_2_ production and cyclooxygenase-2 (COX-2) activity, thereby protecting against bone damage and osteoclast formation [37]. Clinical studies corroborate these findings. Osteoarthritis (OA) is a whole-joint disease associated with chronic pain and disability. Obesity and joint trauma contribute significantly to the progression of OA [38]. In a trial involving 78 osteoarthritis patients, ALA treatment significantly lowered the serum levels of TNF-α, IL-1β, IL-23, IL-6, and IL-17 compared to controls. ALA also downregulated NF-κB and Toll-like receptor 4 (TLR-4) expression in peripheral blood mononuclear cells [39]. In another randomized controlled clinical study including 65 rheumatoid arthritis (RA) patients, the effects of ALA supplementation for eight consecutive weeks were observed. Treatment with ALA did not significantly affect the serum levels of high-sensitivity C-reactive protein (CRP—acute-phase protein), matrix metalloproteinase-3 (MMP-3), or TNF-α compared to the control group [40]. These findings collectively highlight the anti-inflammatory benefits of ALA in managing joint inflammation, though further research is needed to reconcile discrepancies in clinical outcomes.

### 4.3. ALA and Asthma

Asthma is a chronic inflammatory lung disease characterized by coughing, wheezing, shortness of breath, and chest tightness. Its pathogenesis involves multiple immune cells, including neutrophils, lymphocytes, eosinophils, and mast cells, which drive airway inflammation and hyper-responsiveness [41]. In an ovalbumin (OVA)-induced allergic asthma mouse model, ALA demonstrated therapeutic efficacy by significantly reducing the levels of the cytokines IL-4 and IL-5, as well as inflammatory cell counts (eosinophils, neutrophils, and lymphocytes), in bronchoalveolar lavage (BAL) fluid compared to controls. Additionally, ALA decreased periodic acid–Schiff (PAS)-positive cells (indicative of mucus production) in bronchial tissues and lowered intracellular ROS levels in lymphocytes from perihilar lymph nodes [42]. In neonatal mice with OVA-induced asthma, ALA treatment suppressed airway inflammation, as evidenced by an attenuated lung wet-to-dry ratio (a measure of edema), diminished levels of inflammatory mediators (IL-4, IL-5, IL-13, TNF-α), and decreased levels of total IgE and OVA-specific IgE (key drivers of allergic responses). These effects were linked to the inhibition of the NF-κB signaling pathway, further underscoring the anti-inflammatory activity of ALA [43]. Another study highlighted the ability of ALA to alleviate allergic symptoms, such as sneezing and nasal rubbing, while reducing the serum levels of OVA-specific IgE and IgG1. Mechanistically, ALA modulated immune responses by upregulating the Treg-associated transcription factor Foxp3 and anti-inflammatory cytokine IL-10 while downregulating the Th17-associated cytokine IL-17, STAT3, and RORγ. Concurrently, ALA enhanced antioxidant defenses via the activation of the Nrf2/HO-1 signaling pathway and suppressed inflammation by inhibiting the NF-κB/IκB axis [44]. These findings collectively emphasize the therapeutic potential of ALA in asthma management. However, gaps remain in understanding its precise molecular mechanisms, warranting further investigation to optimize its clinical application.

### 4.4. Effects of ALA in Sepsis and Associated Organ Injury

**Figure 2:**
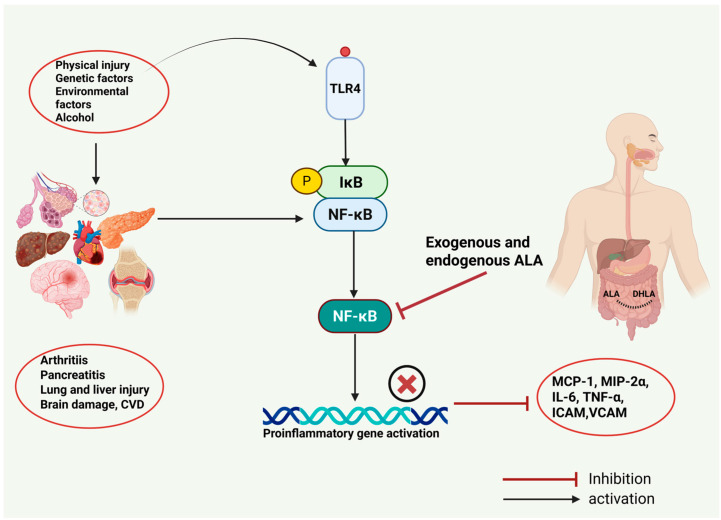
Environmental and genetic factors can contribute to the development of several inflammatory conditions. Exogenous and endogenous ALA can suppress the activation of NF-κB and inhibit the production of proinflammatory mediators.

Sepsis arises from a dysregulated host response to infection, leading to life-threatening organ dysfunction. Its most severe manifestation, septic shock, involves profound circulatory, metabolic, and cellular abnormalities that significantly elevate mortality. Sepsis accounts for nearly 19.7% of deaths due to organ failure [45]. The rising incidence is linked to factors such as antibiotic overuse, immunosuppressive therapies, chemotherapy, and invasive medical procedures [46]. This underscores the urgent need for novel therapeutic strategies to address this critical health burden. Preclinical studies highlight ALA as a promising candidate for mitigating sepsis-related organ injury. In lipopolysaccharide (LPS)-induced endotoxemia in mice, a model where LPS potently stimulates inflammation, the ALA treatment suppressed NF-κB activation, reduced the levels of proinflammatory cytokines (TNF-α, MCP-1) and adhesion molecules (VCAM-1, ICAM-1, E-selectin) in the lungs and heart, and activated the PI3K/Akt pathway. This mechanism preserved endothelial function, attenuated leukocyte adhesion, and improved survival rates compared to untreated controls [47]. In LPS-induced septic mice, intraperitoneal ALA administration demonstrated hepatoprotective effects by lowering the levels of liver enzymes, i.e., alanine aminotransferase (ALT) and aspartate aminotransferase (AST); inhibiting NF-κB and TNF-α; and enhancing mitochondrial function, energy metabolism, and glucocorticoid receptor modulation [48]. The cecal ligation and puncture (CLP) model, a gold-standard sepsis induction method in rodents, further validated ALA’s efficacy. CLP rats treated with ALA exhibited reduced myeloperoxidase activity, hepatic lipid peroxidation, and renal protein carbonylation, alongside increased renal superoxide dismutase activity. Notably, ALA conferred stronger protection against sepsis-induced liver and kidney damage than lung or cardiac injury [49]. In CLP-induced acute lung injury (ALI) models, ALA suppressed NF-κB activation, reduced TNF-α and IL-6 levels, decreased myeloperoxidase and lipid peroxidation, and elevated glutathione (GSH) and superoxide dismutase levels. These effects improved survival rates and attenuated lung tissue damage, including alveolar thickening and hemorrhage [50]. Similarly, ALA alleviated acute kidney injury (AKI) in CLP rats by inhibiting NF-κB; downregulating proinflammatory cytokines (TNF-α, IL-6, IL-1β), iNOS, and HMGB1 expression in renal tissues; and reducing serum blood urea nitrogen (BUN) and creatinine levels [51]. Clinically, an ongoing multicenter trial involving 352 sepsis patients across eight Chinese tertiary hospitals is evaluating ALA (600 mg IV) as an adjuvant therapy. Primary endpoints include 28-day mortality, with secondary outcomes assessing ICU/hospital mortality, AKI incidence, and inflammatory markers. This trial aims to establish clinical evidence for the potential of ALA in mitigating sepsis-driven inflammation and organ dysfunction [52]. Despite these advances, further research is needed to fully elucidate the molecular mechanisms underlying the anti-inflammatory effects of ALA in sepsis. Figure 2 summarizes our current understanding of the potential of ALA in the prevention and treatment of inflammatory conditions.

## 5. ALA and Cardiovascular Diseases

The cardiovascular system, comprising the heart and blood vessels, is susceptible to abnormalities collectively termed cardiovascular diseases (CVDs) [53]. CVDs, particularly heart-related disorders, rank among the leading global causes of mortality [54]. Accumulating evidence highlights ALA as a promising cardioprotective agent, with benefits reported in atherosclerosis, myocardial infarction (MI), and ventricular/atrial pathologies [2]. In in vitro studies using the Langendorff-perfused isolated heart model of ischemia–reperfusion injury, ALA demonstrated cardioprotection by activating ATP-sensitive potassium (K_ATP_) channels and elevating sulfane sulfur levels [55]. In a myocardial infarction (MI) mouse model induced by left anterior descending (LAD) coronary artery ligation, ALA administration reduced infarct size and the serum levels of IL-1β, TNF-α, and creatine kinase-myocardial band (CKMB). Additionally, ALA significantly increased the levels of anti-inflammatory mediators such as IL-10 and TGF-β and improved macrophage M2b polarization. In H9C2 cardiomyocytes and RAW264.7 macrophage cell lines, ALA treatment prevented apoptosis and inflammation by inhibiting the HMGB1/NF-κB signaling pathway [56]. In LPS-induced inflammation in rats, the effects of ALA on ventricular and atrial tissues were evaluated. ALA treatment significantly decreased cardiac edema and the levels of TNF-α, IL-6, thiobarbituric acid reactive substances (TBARS), and hydrogen peroxide (H_2_O_2_). Conversely, ALA increased total sulfhydryl content, total glutathione levels, and superoxide dismutase activity in cardiac tissues [57]. In the LAD coronary artery ligation-induced MI rat model, ALA administration significantly reduced apoptosis, inflammation, and necrosis in cardiomyocytes. Furthermore, ALA decreased the serum levels of CKMB and lactate dehydrogenase (LDH). These cardioprotective effects were linked to the activation of the PI3K/Akt signaling pathway [58]. In a transverse aortic constriction (TAC)-induced heart failure mouse model, ALA treatment showed protective effects by reducing cardiac hypertrophy and enhancing mitochondrial autophagy in wild-type (WT) mice. However, in aldehyde dehydrogenase 2 knockout (ALDH2^−/−^) mice (ALDH2—an enzyme critical for protecting the heart against oxidative damage), no cardioprotective effects of ALA were observed. This finding highlights the essential role of ALDH2 in mediating the cardioprotective effects of ALA [59]. In streptozotocin (STZ)-induced diabetic rats, ALA administration reduced cardiac fibrosis, improved heart function, restored extracellular matrix (ECM) balance, and inhibited collagen deposition. These cardioprotective effects were associated with the suppression of the JNK (c-Jun N-terminal kinase) and p38 MAPK (mitogen-activated protein kinase) signaling pathways [60]. These findings collectively suggest that ALA holds significant potential as a therapeutic agent for the management of cardiovascular diseases.

## 6. ALA and Neurological Disorders

Pathological conditions affecting the nervous system pose significant global health challenges and economic burdens [61]. These disorders are broadly categorized into two classes: neurodegenerative diseases, e.g., Alzheimer’s disease (AD), Parkinson’s disease (PD), and Huntington’s disease (HD), caused by neuronal damage, and neuropsychiatric disorders (e.g., anxiety, migraine, epilepsy, depression), characterized by abnormalities in brain function [62]. Neurodegenerative disorders are often linked to impaired iron metabolism, leading to iron accumulation that exacerbates oxidative stress, induces neuroinflammation, and drives neuronal loss [63]. This process activates microglia, triggering the release of eicosanoids, reactive oxygen species (ROS), cytokines, chemokines, and reactive nitrogen species (RNS). ALA has demonstrated neuroprotective properties attributed to its antioxidant, anti-inflammatory, and metal-chelating activities [64]. In a dapsone-induced neuroinflammation mouse model, ALA suppressed microglial and astrocyte activation; inhibited TNF-α, brain-derived neurotrophic factor (BDNF), and IL-1β production; and enhanced antioxidant defenses [65]. In APP23/PS45 transgenic mice (an AD model), ALA treatment reduced amyloid plaque formation (a hallmark of AD) and improved cognitive function. In vitro studies further revealed that ALA upregulated ADAM10 (A Disintegrin and Metalloproteinase 10), an enzyme critical for the non-amyloidogenic processing of amyloid-β precursor protein (APP). These effects were mediated through autophagy and mitophagy activation [66]. In 6-hydroxydopamine (6-OHDA)-induced PD models (in vivo and in vitro), ALA administration reduced iron accumulation, ROS levels, and neuronal loss while enhancing antioxidant activity [22]. Similarly, in 1-methyl-4-phenyl-1,2,3,6-tetrahydropyridine (MPTP)-induced neuroinflammation, ALA improved motor function, inhibited microglial activation in the spinal cord and substantia nigra (SN), and suppressed NF-κB, iNOS, and TNF-α expression [67]. In the LPS-induced PD mouse model, ALA prevented α-synuclein accumulation in the SN, mitigated neuronal damage, and inhibited NF-κB activation and proinflammatory mediator expression [68]. In a 3-nitropropionic acid (3-NP)-induced HD rat model, ALA ameliorated mitochondrial dysfunction, oxidative damage, and spatial memory deficits [69]. Clinical studies also support the neuroprotective potential of ALA. A trial involving 24 multiple sclerosis (MS) patients demonstrated that ALA reduced IL-6 and IL-17 production, increased IL-10 synthesis, and modulated neuroinflammation via the cAMP/PKA signaling pathway [70]. Another randomized controlled trial with 52 MS patients showed that ALA significantly decreased the serum levels of proinflammatory mediators, including ICAM-1, IL-4, TGF-β, and IFN-γ, compared to controls [71]. These findings collectively establish the therapeutic potential of ALA in managing neurological disorders.

## 7. ALA and Formation of H_2_S

While ALA is well established as an antioxidant, emerging evidence highlights its anti-inflammatory properties, though the molecular mechanisms underlying these effects remain incompletely understood. Recent studies propose that ALA’s therapeutic benefits are linked to sulfane sulfur metabolism [72]. In rat liver homogenates, ALA administration generates DHLA, which promotes hydrogen sulfide (H_2_S) release via sulfane sulfur metabolism [73]. H_2_S, a gasotransmitter characterized by its pungent odor reminiscent of rotten eggs, is the third member of the gasotransmitter family, following nitric oxide (NO) and carbon monoxide (CO). It is primarily synthesized by three enzymes: cystathionine γ-lyase (CSE), cystathionine β-synthase (CBS), and 3-mercaptopyruvate sulfurtransferase (3-MST) [74]. H_2_S plays a critical role in various pathological conditions and is considered a therapeutic target at concentrations ranging from low nanomolar to mid-micromolar levels. In a zymosan-induced peritonitis mouse model, ALA significantly reduced neutrophil infiltration and vascular permeability. These anti-inflammatory effects were found to be mediated by the release of H_2_S via sulfane sulfur metabolism [75]. Another study in a type 2 diabetes mellitus (T2DM) rat model demonstrated that oral ALA administration markedly upregulated the expression of 3-MST and CSE, increased sulfane sulfur levels, and enhanced H_2_S formation. This intervention provided significant protection against inflammation, liver sinusoidal enlargement, and hepatocellular vacuolation, thereby maintaining insulin secretion, lipid metabolism, and glucose homeostasis. In contrast, treatment with DL-propargylglycine (PAG), a CSE inhibitor, exacerbated T2DM-induced liver injury, leading to severe hepatocellular vacuolation, heightened oxidative stress, and amplified inflammation [76]. A clinical study involving 101 individuals with T2DM revealed that two weeks of ALA supplementation, when combined with conventional anti-diabetic therapy, significantly improved endothelial dysfunction by elevating H_2_S levels [77]. These findings collectively suggest that the protective effects of ALA are closely tied to the H_2_S signaling pathway. In a carrageenan-induced paw edema mouse model, the intraperitoneal administration of ALA significantly reduced paw edema, exhibiting anti-inflammatory effects comparable to those of indomethacin, a standard anti-inflammatory drug. However, the co-administration of glibenclamide, a compound that blocks K_ATP_ channels, which are key mediators of H_2_S signaling, diminished the therapeutic effects of ALA and exacerbated paw edema [78]. Similarly, in isolated rat hearts subjected to ischemia–reperfusion injury, ALA treatment exerted cardioprotective effects by increasing sulfane sulfur levels and H_2_S formation, which prevented post-reperfusion arrhythmias and hypoxia-induced cell death. Again, glibenclamide administration inhibited these cardioprotective effects, underscoring the hypothesis that the therapeutic actions of ALA extend beyond its antioxidant properties and are critically dependent on H_2_S signaling [55].

**Figure 3:**
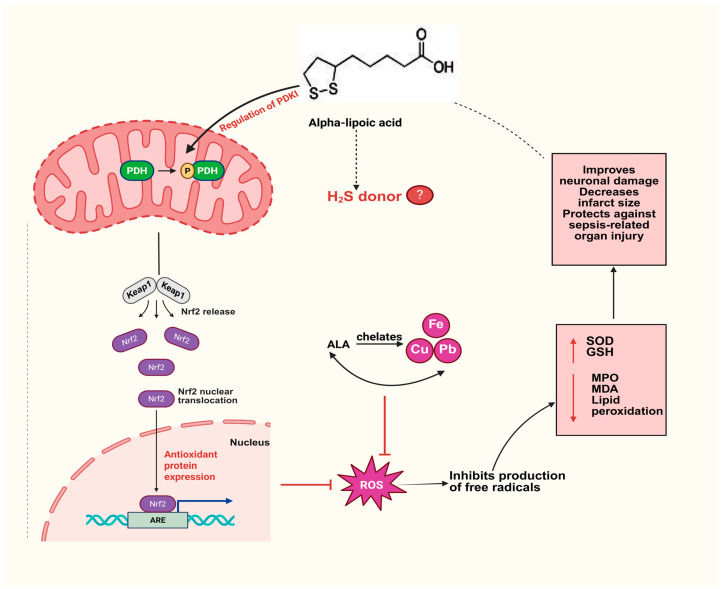
ALA can modulate the activation of the Nrf2/ARE pathway, which inhibits the production of free radicals and decreases MPO, MDA, and lipid peroxidation. It also improves neuronal damage, decreases infarct size, and protects against sepsis-related organ injury.

Figure 3 summarizes our current understanding of the molecular mechanisms by which ALA can act in the prevention and treatment of different disease conditions.

Despite these promising findings, there remains a significant gap in understanding the precise molecular mechanisms by which ALA influences H_2_S signaling. Further research is essential to fully elucidate these pathways and their implications for therapeutic applications.

The role of ALA in different disease conditions is summarized in Table 1.

## 8. Conclusions

ALA is a versatile therapeutic agent with significant potential in the management of inflammatory diseases, cardiovascular disorders, and neurological conditions. The antioxidant and anti-inflammatory properties of ALA make it an effective agent against oxidative stress-induced damage. Beyond its well-established antioxidant properties, emerging evidence suggests that ALA may exert additional protective effects by modulating hydrogen sulfide (H_2_S) signaling, further enhancing its therapeutic potential. These multifactorial properties shed light on ALA as a promising therapeutic approach in the treatment of chronic diseases driven by inflammation and oxidative stress.

## 9. Future Perspectives

Despite a substantial body of evidence suggesting the anti-inflammatory and antioxidant properties of ALA, several questions, such as those regarding the optimal dose and dosing regimen of ALA, remain unanswered. A deeper understanding of the H_2_S-dependent mechanism of ALA, regarding precise molecular targets within the H_2_S biosynthesis and signaling cascade of ALA, also needs to be elucidated. ALA has, however, exhibited a good safety profile in short-term preclinical and clinical studies at different therapeutic ranges, which is promising. Further research is needed in order to investigate the benefits (as well as potential risks) of the long-term use of ALA in the clinic.

## Figures and Tables

**Table 1:** Role of ALA in different diseases.

**Disease**	**Mechanism of Action**	**Disease Model**	**ALA Dose and Administration Route**	**Effects**	**Reference**
Acute pancreatitis	Not completely understood	CKK-octapeptide-induced AP in rats (in vivo)	ALA was administered intraperitoneally at 1 mg/kg	Reduced levels of serum lipases and amylases and pancreatic weight–body weight ratio	[31]
	Antioxidant and anti-inflammatory effects	Cerulein-induced AP in rats (in vivo)	ALA was administered intraperitoneally at dose of 100 mg/kg	Increased glutathione levels and decreased serum amylase and lipase levels and MDA and MPO activities Reduced necrosis and attenuated pancreatic tissue damage	[32]
	Activation of PPAR-γ signaling pathway	Cerulein/resistin-induced oxidative stress and inflammation in pancreatic acinar AR42J cells (in vitro)	Prophylactic treatment of AR42J cells with α-lipoic acid (at dose of 2 or 5 µM)	Decreased IL-6 expression and ROS production Increased expression of HO-1 and catalase	[33]
Joint inflammation	Suppression of NF-κB signaling pathway	Collagen-induced arthritis (CIA) mouse model (in vivo)	160 to 800 mg/kg per day (fed ALA diet) and 10 or 100 mg/kg (intraperitoneally)	Decreased production of IL-6, TNF-α, and IL-1β Decreased bone destruction and inhibition of production of TRAP-positive osteoclasts	[35,36]
	Suppression of receptor activator of nuclear factor kappa-B ligand (RANKL) expression	Osteoclastic bone damage with inflammation in mice and osteoclast cultures (in vivo and in vitro)	10 µM and 50 µM (in vitro) and 25 mg/kg was administered intraperitoneally	Decreased PGE_2_ production and COX-2 activity	[37]
	Suppression of NF-κB and TLR-4 expression	78 osteoarthritis patients (clinical study)	Oral administration of 0.6 g of ALA once	Decreased levels of serum TNF-α, IL-1β, IL-23, IL-6, and IL-17	[39]
Asthma	Antioxidant activities	OVA-induced allergic asthma mouse model (in vivo)	Different concentrations of ALA, i.e., 0%, 0.125%, 0.25%, 0.5%, and 1%, were administered through diet	Decreased intracellular ROS levels in lymphocytes, IL-4 and IL-5, eosinophils, neutrophils, and lymphocytes in BAL	[42]
	Suppression of NF-κB signaling pathway	OVA-induced neonatal mice (in vivo)	1% ALA was administered orally, mixed with mouse chow	Suppressed airway inflammation; lower wet-to-dry ratio in lungs; decreased levels of IL-4, IL-5, IL-13, and TNF-α; and lower levels of total IgE	[43]
	Activation of Nrf2/HO-1 and inhibition of NF-κB signaling pathway	(OVA)-induced allergic rhinitis (AR) mouse model (in vivo)	ALA was administered orally at various doses (2, 10, and 50 mg/kg)	Upregulation of Foxp3 and IL-10 Downregulation of IL-17, STAT3, and RORγ	[44]
Sepsis-related organ injury	Activation of PI3K/Akt signaling pathway and suppression of NF-κB activity	LPS-induced endotoxemia mouse model (in vivo)	ALA was administered intraperitoneally at 100 mg/kg	Decreased levels of TNF-α, MCP-1, VCAM-1, ICAM-1, and E-selectin	[47]
	Suppression of NF-κB activity	LPS-induced sepsis mouse model (in vivo)	ALA was administered intraperitoneally at 100 mg/kg	Acute liver injury Decreased levels of liver enzymes (ALT and AST) and TNF-α Improved mitochondrial function	[48]
	Antioxidant activities	CLP-induced sepsis model in rats (in vivo)	ALA was administered at dose of 200 mg/kg	Sepsis-associated acute kidney and liver injury Decreased MPO activity, lipid peroxidation in liver, and protein carbonylation in kidneys Increased SOD activity in kidneys	[49]
	Suppression of NF-κB activation	CLP-induced sepsis model in rats (in vivo)	ALA was administered by oral gavage at dose of 200 mg/kg	Acute lung injury Decreased TNF-α, IL-6, and MPO activity levels Increased GSH and SOD activities	[50]
Cardiovascular diseases	Inhibition of NF-κB signaling pathway	LAD coronary artery ligation mouse model (in vivo)	ALA was administered intraperitoneally at dose of 30 mg/kg	Decreased infarct size and serum IL-1β, TNF-α, and CKMB levels	[56]
	Suppression of JNK and p38 MAPK pathways	Streptozotocin-induced diabetic rats (in vivo)	ALA was administered intraperitoneally at dose of 100 mg/kg	Reduced cardiac fibrosis, improved heart function, restored ECM balance, and inhibited collagen deposition	[60]
	Activation of PI3K/Akt pathway	LAD coronary artery ligation-induced MI rat model (in vivo)	ALA was administered by tail vein injection at dose of 15 mg/kg	Decreased serum CKMB levels, LDH apoptosis, inflammation, and necrosis in cardiomyocytes	[58]
Neurological diseases	Activation of autophagy and mitophagy	APP23/PS45 transgenic mouse model (in vivo)	ALA was administered intraperitoneally at dose of 5 mg/kg	Reduced amyloid plaque formation in brain and improved cognitive functions	[66]
	Inhibition of NF-κB, iNOS, and TNF-α expression	MPTP-induced neuroinflammation mouse model (in vivo)	ALA was administered intraperitoneally at dose of 50 mg/kg	Improved motor function and prevented activation of microglia in spinal cord and SN	[67]
	cAMP/PKA signaling pathway	24 subjects suffering from MS (clinical study)	ALA was administered orally in form of capsules at dose of 600 mg	Markedly reduced IL-6 and IL-17 production and increased IL-10 synthesis	[70]
